# Hyperammonemia increases the release of pathological extracellular vesicles from monocytes by impairing lysosomal function and autophagy through the TNFα–cAMP–PKA–LC3 pathway

**DOI:** 10.3389/fimmu.2025.1724800

**Published:** 2026-01-12

**Authors:** Maria A. Pedrosa, Paula Izquierdo-Altarejos, Marta Llansola, Vicente Felipo

**Affiliations:** 1 Laboratory of Neurobiology, Centro de Investigación Príncipe Felipe, Valencia, Spain; 2 INCLIVA Instituto de Investigación Sanitaria, Valencia, Spain

**Keywords:** CD4^+^ T lymphocytes, extracellular vesicles, hyperammonemia, lysosomal-autophagy dysfunction, minimal hepatic encephalopathy, monocytes, neuroinflammation, TNFα

## Abstract

**Background:**

Patients with liver cirrhosis may show minimal hepatic encephalopathy (MHE) triggered by a shift in peripheral inflammation. A main mechanism by which peripheral alterations are transmitted to the brain is the infiltration of extracellular vesicles (EV). Hyperammonemic rats are a model of MHE that reproduces cognitive impairment. Injection of EV from plasma or peripheral blood mononuclear cells (PBMC) of hyperammonemic rats to normal rats induces neuroinflammation, alterations in neurotransmission, and cognitive impairment. PBMC contain different cell types. The aims were 1) to identify which cell type produces the pathological EV in hyperammonemic rats; 2) to identify the mechanisms by which hyperammonemia increases EV release from monocytes and induces the formation of pathological EV; and 3) to analyze the role of TNFα and PKA in these mechanisms.

**Methods:**

EV were isolated from primary cultures of CD4^+^ lymphocytes or monocytes from control or hyperammonemic rats and added to hippocampal slices from control rats to assess induction of neuroinflammation and changes in neurotransmission. To assess the role of TNFα and protein kinase A (PKA) in the production of pathological EV by monocytes from hyperammonemic rats, we blocked TNFα with anti-TNFα or inhibited PKA. Lysosomal-autophagy dysfunction was assessed with LysoTracker and by analyzing cathepsin L, LAMP2, and LC3.

**Results:**

In hyperammonemic rats, monocytes but not CD4^+^ lymphocytes release pathological EV. Hyperammonemia increases the EV release by monocytes and their content of TNFR1 and TNFα. These EV induce activation of glia and of the TNFα–TNFR1–S1PR2−IL-1β–CCL2–BDNF–TrkB pathway and alterations in membrane expression of NMDA and AMPA receptors in hippocampal slices from control rats. Hyperammonemia increases TNFα levels in monocytes, which increases cAMP and PKA activity and reduces LC3 content. This leads to autophagy–lysosome dysfunction, with altered LC3, cathepsin L, and LAMP2 content and pH that increases the release of EV and their TNFR1 and TNFα content. All these changes are reversed by blocking TNFα with anti-TNFα or inhibiting PKA with an inhibitor.

**Conclusions:**

These data unveil that monocytes produce the pathological EV in hyperammonemia and the underlying mechanisms and provide the bases for new treatments to improve cognitive and motor function in hyperammonemia and MHE.

## 1 Introduction

Patients with liver cirrhosis may show MHE with mild cognitive impairment, attention deficits, and motor incoordination which reduce their quality of life and life span (1–7). To develop useful treatments for MHE, it is necessary to understand the mechanisms triggering it. Rats with chronic hyperammonemia (HA) reproduce many of the alterations in cognitive and motor function present in MHE patients and are a good model to investigate the mechanisms and treatments of MHE (8–11). A shift in peripheral inflammation to a more pro-inflammatory state with activation of Th17 CD4^+^ lymphocytes plays a key role in triggering the appearance on MHE in cirrhotic patients (12, 13) and in animal models (14–16). These changes in peripheral inflammation are transmitted to the brain to trigger neuroinflammation, alterations in neurotransmission, and MHE (10, 11, 17).

Several mechanisms may contribute to the transmission to the brain of the peripheral alterations, including infiltration into the brain of cells from the peripheral immune system (18–22) or infiltration of extracellular vesicles from the blood (23–27). EV are small vesicles released by most cell types which play an important role in intercellular communication, especially in the immune system (27–30). EV may cross the blood–brain barrier and transmit pathological effects to the brain. For example, injection to mice of EV from serum or cerebrospinal fluid of Parkinson’s disease patients induce motor symptoms and pathology in the mice (31, 32).

We have previously shown that hyperammonemic rats have increased levels of EV in plasma and that these EV show altered protein content including increased levels of TNFα and tumor necrosis factor alpha receptor type 1 (TNFR1). Intravenous injection of EV from plasma of hyperammonemic rats to normal rats induces neuroinflammation and alterations in glutamatergic neurotransmission in hippocampus and cognitive impairment (26). These results show that plasma of hyperammonemic rats contain pathological EV. Identifying the mechanisms by which these pathological EV are generated will help to develop new treatments to reverse MHE.

EV present in plasma can come from different organs, tissues, or cell types, but most EV come from cells of the immune system (33–35).

We therefore assessed if pathological EV in plasma of hyperammonemic rats are generated by PBMC. We cultured PBMC from control and hyperammonemic rats and isolated the EV released to the culture medium. Injection to normal rats of EV released by PBMC from hyperammonemic rats (HA-PBMC-EV), but not from control rats, induced neuroinflammation and alterations in glutamatergic neurotransmission in hippocampus and cognitive impairment (36). This indicates that immune cells are responsible for the generation of the pathological EV in hyperammonemic rats. Addition of HA-PBMC-EV ex vivo to hippocampal slices from control rats also induces neuroinflammation, glial activation, and alterations in neurotransmission similar to those observed in rats *in vivo*. Moreover, this *ex vivo* system allows to identify in detail the mechanisms involved in the pathological effects of the EV and design systems to prevent their toxicity. We showed that blocking TNFα in the HA-PBMC-EV prevents their effects on neuroinflammation and neurotransmission (36).

PBMC contain different cell types including CD4^+^ lymphocytes and monocytes. Identifying which specific cell type produces the pathological EV in hyperammonemia would help to design new treatments for MHE.

The first aim of this work was to identify which cell type produces the pathological EV in hyperammonemic rats. Cirrhotic patients with MHE show activated pro-inflammatory CD4^+^ lymphocytes and monocytes (12, 13). We therefore hypothesized that these cell types could contribute to the production of the pathological EV in hyperammonemic rats. We therefore prepared primary cultures of CD4^+^ lymphocytes or of monocytes from control and hyperammonemic rats and isolated the EV released by these cell types. We added the EV from control or hyperammonemic rats to hippocampal slices from control rats and analyzed the effects on neuroinflammation and neurotransmission. We found that EV from monocytes (HA-M-EV) but not from CD4^+^ lymphocytes (HA-CD4-EV) of hyperammonemic rats induce the pathological effects.

Once having identified that monocytes produce pathological EV, the next aims were a) to identify the mechanisms involved in the production by HA monocytes of pathological EV and b) to identify the mechanisms by which hyperammonemia increases the number of EV produced by the monocytes.

We have previously shown that pathological EV in plasma of hyperammonemic rats have an increased content of TNFα and of the TNFα receptor TNFR1, which are involved in the mechanisms by which EV induce cognitive and motor impairment in hyperammonemic rats (26). Islam et al. (37) showed that cAMP induces the release of TNFR1 in exosome-like vesicles via a PKA-dependent mechanism. We therefore hypothesized that enhanced activation of PKA in HA monocytes would mediate the release of pathological EV. To assess this hypothesis, we tested if inhibiting PKA in the primary cultures of monocytes of hyperammonemic rats prevents the production of pathological EV. We also hypothesized that the enhanced PKA activity in monocytes from hyperammonemic rats would be due to a TNFα-induced increase in cAMP. We assessed if the increase in cAMP and the production of pathological EV are prevented by treating the monocytes with anti-TNFα.

It has been reported that lysosomal dysfunction enhances EV release (38–40). In cultured astrocytes, pathological α-synuclein accumulation induces an increase in EV release, which was attributed to altered lysosomal function, with reduced cathepsin L levels (38). We therefore assessed if the increase in the number of EV released in hyperammonemia could be due to lysosomal dysfunction. We also assessed if lysosomal dysfunction in monocytes from hyperammonemic rats is a consequence of enhanced cAMP-PKA system and or TNFα levels.

## 2 Methods

### 2.1 Rats with chronic hyperammonemia

Male Wistar rats (Charles River Laboratories) were fed with an ammonium containing diet (pellets with 30% of ammonium acetate), as described in (41). Rats after 4–5 weeks of hyperammonemia and age-matching controls were used. Different rats were used for isolation of CD4^+^ lymphocytes or monocytes and for the *ex vivo* experiments. All the experiments were approved by the Comite de Experimentación y Bienestar Animal (CEBA) of Príncipe Felipe Research Center and by Conselleria de Agricultura of Generalitat Valenciana and were performed in accordance with guidelines of the Directive of the European Commission (2010/63/EU) for care and management of experimental animals.

### 2.2 Experimental design

**Figure 1:**
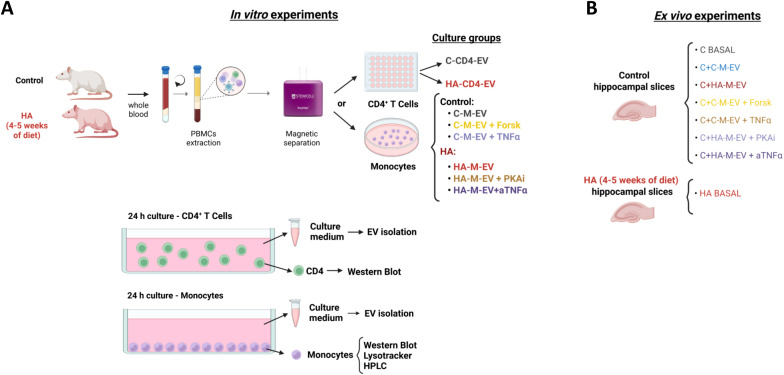
Experimental design. **(A)** For *in vitro* experiments, peripheral blood mononuclear cells (PBMCs) were isolated from whole blood by density gradient separation. CD4^+^ T lymphocytes and monocytes were isolated using EasySep commercial negative selection kits and cultured for 24 h in the following groups: CD4^+^ lymphocytes from control and hyperammonemic rats (C-CD4-EV and HA-CD4-EV, respectively), monocytes from control and hyperammonemic rats (C-M-EV and HA-M-EV, respectively), monocytes from hyperammonemic rats treated with H89, a protein kinase A inhibitor (PKAi) (HA-M-EV + PKAi) or anti-TNFα (HA-M-EV + aTNFα), and control monocytes treated with forskolin, an activator of adenylate cyclase, that increases cAMP and activates PKA (C-M-EV + Forsk) or with recombinant TNFα (C-M-EV + TNFα). Cultured cells were collected for their respective analyses, and EV were isolated from the culture media of the different experimental groups **(B)** For *ex vivo* experiments, EV from the different groups (C-CD4-EV, HA-CD4-EV, C-M-EV, HA-M-EV, HA-M-EV + PKAi, HA-M-EV + aTNFα, C-M-EV + Forsk, C-M-EV + TNFα) were added to hippocampal slices from control rats. Slices from control (C BASAL) and hyperammonemic (HA BASAL) rats without EV incubation were included as reference. After the incubation, slices were processed for analysis.

The study design includes *in vitro* and *ex vivo* experiments and is summarized in **Figure 1**. First, peripheral CD4^+^ lymphocytes or monocytes were isolated and cultured from control and hyperammonemic rats. Primary cultures of monocytes from hyperammonemic rats (HA-monocytes) were seeded in medium containing H89, a protein kinase A inhibitor (PKAi) (HA-M-EV+PKAi) or anti-TNFα (HA-M-EV+aTNFα) to assess the contribution of PKA and TNFα in the monocytes to the effects of the HA-M-EV. With the same aim, primary cultures of monocytes from control rats (C-monocytes) were treated with forskolin, an activator of adenylate cyclase that increases cAMP and activates PKA (C-M-EV+Forsk) or with recombinant TNFα (C-M-EV+TNFα) (**Figure 1A**). EV were isolated from the culture medium of CD4^+^ lymphocyte and monocytes from control rats (C-CD4-EV and C-M-EV, respectively) and hyperammonemic rats (HA-CD4-EV and HA-M-EV, respectively).

In the *ex vivo* experiments (**Figure 1B**), hippocampal slices from control rats were incubated with HA-CD4-EV, C-CD4-EV, HA-M-EV, HA-M-EV+PKAi, HA-M-EV+aTNFα, C-M-EV, C-M-EV+Forsk, or C-M-EV+TNFα. As a reference group for the pathological effects in the mechanisms studies, hippocampal slices from hyperammonemic rats (HA) were also analyzed.

### 2.3 CD4^+^ lymphocytes and monocyte isolation

PBMCs were isolated from whole blood by density gradient separation with Lymphoprep (StemCell Technologies, #18060) as in (36). Rats were sacrificed by CO_2_ inhalation, and blood was collected from the cava vein, diluted 1:1 with 0.9% NaCl, and centrifuged in Lymphoprep-containing tubes at 400 g for 30 min. After washing with 0.9% NaCl and RPMI 1640 medium (Gibco, #11530586), cells were counted and resuspended in EasySep Buffer (StemCell Technologies, #20144) before further separation. CD4^+^ T lymphochytes and monocytes were isolated from different rats using EasySep commercial negative selection kits following the manufacturer’s instructions (#19642 and #19609, respectively, from StemCell Technologies). All donor rats were age-matched and batch-matched. Briefly, PBMCs were incubated with the isolation antibody cocktail (50 µl/mL) for 10 min at RT and then with the magnetic beads (25 µL/mL) provided in the kit for 3 min at RT into an EasySep magnet (StemCell Technologies, #18000). The enriched cell suspension was then poured into a new tube, and the isolated cells were counted.

### 2.4 Cell cultures

CD4^+^ lymphocytes were resuspended in X-VIVO 20 serum-free medium (Lonza, #04-448Q) containing 1% penicillin/streptomycin (Gibco, #15140122) at 10^6^ cells/mL and cultured in 48-well plates (500,000 cells/well) precoated with 1 µg/mL of anti-CD3 (BD Biosciences, #554829) as in (42).

Monocytes were resuspended in X-VIVO 20 serum-free medium (Lonza, #04-448Q) containing 1% penicillin/streptomycin (Gibco, #15140122) at 250,000 cells/mL and cultured in 35×10 mm culture dishes (Corning, #430165). To study the effects of TNFα and cAMP/PKA signaling, monocytes from hyperammonemic rats were incubated with an anti-TNFα antibody (aTNFα, 1µg/mL; R&D Systems, #AF-510-NA) or 10 µM of H-89 (Sigma, #371963), a PKA inhibitor (PKAi). Monocytes from control rats were incubated with 10 ng/mL rat recombinant TNFα (Abcam, #ab9756) or 20 µM forskolin (Sigma, F6886). The dose of the treatments was chosen based on previous studies: rat recombinant TNFα as in (26); forskolin as in (43); and H-89 as in (44). The dose of the anti-TNFα antibody was based on the neutralization effect according to the manufacturer’s instructions. DMSO was included in the corresponding control condition for forskolin treatments. Vehicle controls were not required for the other treatments, as they were diluted in distilled water.

All CD4^+^ lymphocytes and monocytes cultures were incubated at 37°C with 5% CO_2_ for 24 h.

### 2.5 Extracellular vesicle isolation and characterization

EV were isolated from cell culture media as in (36). First, medium was centrifuged at 2,000 g, 10 min, 4°C, and 10,000 g, 30 min, 4°C. Then, the supernatant was ultra-centrifuged at 110,000 g, 1 h, 4°C, and the final pellet containing the EV was resuspended in 100 µL of filtered PBS.

Transmission electron microscopy (TEM) was used to visualize and assess the morphology and size of the EV. Isolated vesicles were observed after negative staining with uranyl acetate solution 1% w/v using a FEI Tecnai G2 Spirit Transmission Electron Microscope, and images were acquired with an Olympus digital camera.

Nanoparticle tracking analysis (NTA) was used to measure the size and quantity of isolated vesicles. EV were diluted 1:50 and analyzed with a NanoSight NS300 system (Malvern Panalytical). For each sample, five videos of 30 s were acquired, and they were analyzed with NTA 3.2 Dev Build 3.2.16 software.

### 2.6 *Ex vivo* experiments

To study the effects of the EV on neuroinflammation and glutamatergic neurotransmission in the hippocampus, EV from the different cultures and conditions were added to hippocampal slices from control rats *ex vivo* as in (36). Control and hyperammonemic rats after 4–5 weeks of HA were sacrificed by decapitation. The hippocampi were dissected and immersed in ice-cold Krebs buffer (NaCl 119 mM, NaHCO_3_ 26.2 mM, glucose 11 mM, KCl 2.5 mN, CaCl_2_ 2.5 mM, KH_2_PO_4–_1 mM aerated with 95% O_2_, and 5% CO_2_ at pH 7.4). Hippocampal slices of 400 μm were obtained with a manual chopper and incubated in the wells of a perfusion system (Campden Instruments) for 15 min at 35.5°C in Krebs buffer for stabilization. Once stabilized, hippocampal slices from control rats were incubated during 30 min at 35.5°C with 5 µg of protein of the EV isolated from the culture medium of CD4^+^ lymphocytes from control and HA rats, or with the six types of EV from monocytes described above: HA-M-EV, HA-M-EV + PKAi, HA-M-EV + aTNFα, C-M-EV, C-M-EV + Forsk, or C-M-EV + TNFα. Slices from control rats incubated in Krebs buffer without EV were included as basal condition. As a reference group for the pathological effects on the mechanistic studies, hippocampal slices from hyperammonemic rats incubated with Krebs buffer were also analyzed.

### 2.7 Microglia and astrocyte activation analysis by immunohistochemistry

*Tissue processing and staining*. After the *ex vivo* incubation, hippocampal slices were fixed by immersion in 4% paraformaldehyde in 0.1 M phosphate buffer and embedded in paraffin. 5 µm sections were obtained with a microtome, mounted into coated slides, and used for immunohistochemistry staining. Samples were incubated for 15 min with 3% H_2_O_2_ to block endogenous peroxidase activity and with normal goat serum for 1 h to avoid unspecific interactions. Then, slides were incubated overnight at 4°C with the primary antibodies against Iba1 (1:300, Wako, #019-19741) to stain the microglia and GFAP (1:300, Sigma, #G3893) to stain astrocytes. After washing, biotinylated secondary antibodies were added and the slides were incubated for 1 h at room temperature. The antibodies were goat anti-rabbit (#BA-1000) for Iba1 staining and goat anti-mouse (#BA-9200) for GFAP staining, both at 1:200 dilution and from Vector Laboratories. VECTASTAIN Elite ABC kit (Vector Laboratories, #PK-6100) was used to amplify the signal, and DAB Substrate kit (Abcam, #ab64238) was employed to obtain the chromogenic staining. Finally, samples were incubated with Mayer’s hematoxylin (DAKO, #MHS32) to better visualize the hippocampal structures and the slides were cover-slipped for further analysis.

*Image acquisition and analysis.* Slides were scanned using a 40× objective with an Aperio Versa system (Leica Biosystems). From these scans, eight images per rat and antibody were acquired with the software ImageScope64.

### 2.8 Analysis of microglial TNFα content by double immunofluorescence and confocal microscopy

Hippocampal slices were washed in 0.1 M phosphate buffer and blocked with normal serum from the same species as the secondary antibody before being incubated (4°C, overnight) with a primary antibody. Double immunofluorescences were performed to analyze TNFα (1:500, RD Systems, #AF-510-NA) in Iba1^+^ cells (Iba1, 1:300, 019-19741, Wako).

The sections were observed under a confocal microscope (Leica SP8 HyVolution II) and imaged with a 63× objective lens. Z-stack imaging was obtained from five (63× magnification) images of hippocampus slices using constant microscopy parameters and similar laser intensity. For analysis of TNFα in Iba^+^ cells, the cytosol of each positive cell was manually outlined using the freehand selection tool of LasX, and the mean intensity of each cell was analyzed with ImageJ. The mean of all images of each animal was calculated.

### 2.9 Analysis of protein content by Western blot

Homogenates from hippocampal slices from the *ex vivo* experiments (see above) or homogenates from CD4^+^ and monocyte primary cultures and EV were subjected to electrophoresis and immunoblotting as in (45). Primary antibodies used were against Alix (1:500, ProteinTech, #12422-1-AP), BDNF (brain-derived neurotrophic factor, 1:1,000, Invitrogen, #OSB00017W), Cathepsin-L (1:200, R&D #AF1515), CCL2 (1:2,000, Proteintech, #66272-1), Flotillin-2 (1:500, Invitrogen, #PA5-17178), IL-1β (1:500, RD Systems, #AF-501-NA), LAMP2 (1:200, Novus Biologicals, #NB300-591), LC3 (microtubule-associated protein 1A/1B-light chain 3, 1:1,000, Novus Biologicals, NB100-2220), S1PR2 (Sphingosine-1-Phosphate Receptor Type 2, 1:1,000, Proteintech, #21180-1-AP), TNFα (1:500, RD Systems, #AF-510-NA), TNFR1 (1:1,000, Abcam, #ab19139), TrkB (tyrosine receptor kinase B, 1:500, Abcam, #ab18987), and β-actin (1:5000, Abcam, #ab6276) or GAPDH (glyceraldehyde-3-phosphate dehydrogenase, 1:5,000, Millipore, #MAB374) as loading control. Secondary antibodies were anti-rabbit, anti-mouse, anti-goat IgG conjugated with alkaline phosphatase (1:4,000, Sigma, #A8025, A3562 and A7650, respectively) or anti-rabbit, anti-mouse, anti-goat IgG conjugated with HRP (1:4,000, Sigma, #A6154, #A4416, #A31460 respectively). Membranes were scanned using the Scanjet 5300C (Hewlett-Packard) for alkaline phosphatase and Amersham ImageQuant 800 system for HRP. Band intensities were quantified using Alpha Imager 2200 version 3.1.3 (Alpha Innotech Corporation). Results were expressed as percentage of the control group.

### 2.10 Analysis of membrane expression of receptors by cross-linking with BS3

Membrane expression of receptors was analyzed in hippocampal slices from the *ex vivo* experiments (see above) by cross-linking with BS3 (bis(sulfosuccinimidyl)suberate, Thermo Fisher, #21580) as in (46). Slices were incubated with or without BS3 (2 mM) for 30 min at 4°C with gentle shaking. Cross-linking was terminated by quenching the reaction with 100 mM glycine (10 min, 4°C). The hippocampal slices were homogenized and analyzed by Western blot as described above, using the following primary antibodies: GluaA1 (Glutamate A1 Subunit of Ampa Receptors, 1:1,000, Millipore, #04-855), GluA2 (Glutamate A2 Subunit of AMPA Receptors, 1:2,000, Proteintech, #11994-1-AP), IL-1R (Interleukin 1-Beta Receptor, 1:500, Abcam, AB106278), NR2A (2A subunit of NMDA receptors, 1:1,000, Millipore, #04-901), NR2B (2B subunit of NMDA receptors, 1:1,000, Millipore, #06-600), TNFR1 (1:1,000, Abcam, #ab19139), TrkB (1:500, Abcam, #ab18987), and S1PR2 (1:1,000, Proteintech, #21180-1-AP). The membrane expression of receptors was calculated as the difference between the intensity of the bands without BS3 (total protein) and with BS3 (non-membrane protein) as in (46).

### 2.11 LysoTracker deep red staining

Monocytes were seeded onto glass coverslips precoated with 0.1% poly-L-lysine hydrobromide (Sigma, #P6282) that were placed inside the culture wells. After 24 h for culture, the culture medium was replaced with fresh medium containing 75 nM LysoTracker Deep Red (Thermo Fisher, #L12492) for 60 min at 37°C. Cells were then fixed with 4% paraformaldehyde at room temperature, and the coverslips were mounted onto glass slides for confocal imaging (Leica SP8 HyVolution II, 63× objective).

### 2.12 Determination of cAMP by LC-MS

Cells (25.000 cells/well) were seeded in 24-well plates and scraped with 100 µL of 0.4 M perchloric acid. After centrifugation for 15 min at 17.000g and 4°C, 50 µL of the supernatant was injected in the LC-MS system. The corresponding pellet was resuspended in 100 µL of 0.1 M NaOH, and protein content was analyzed with the bicinchoninic acid (BCA) method (Pierce, Thermo Fisher Scientific). The procedure described by (47) was used for LC-MS analysis of cAMP with some modifications. Chromatographic separation was carried out at room temperature using the Luna Omega Polar C18 (OOD-4760-AN) 100*2.1 mm 3 µm (100 A) column from Phenomenex. Mobile phase A consisted of 0.1% formic acid in water and mobile phase B was 100% acetonitrile. The following gradient elution profile was applied at a flow rate of 0.4 mL/min: 0.00 min: 90% A, 0.50 min: 90% A, 1.00 min: 10% A, 2.20 min: 10% A, 2.30 min: 90% A, 3.50 min: 90% A. Eluates were detected with the QTRAP 4500 triple quadrupole mass spectrometer (AB Sciex, Ontario, Canada) in the positive electrospray ionization (ESI) mode by multiple reaction monitoring (MRM) in the following conditions: curtain gas 20 psi, GS1–45 psi, GS2–60 psi, ion spray voltage 5.500 V, T^a^ 600°C, entrance potential (EP): 10, declustering potential (DP): 91, collision energy (CE): 35, collision exit potential (CXP): 10. The 330.08 to 136.10 transition was used for quantification of cAMP. Standard solutions of cAMP were prepared in 0.4 M of perchloric acid from 0.05 to 100 nM. Quantification of cAMP concentration in the samples was carried out with the Analyst software.

### 2.13 Statistical analysis

Data are expressed as mean ± standard error of the mean (SEM). All statistical analyses were performed using GraphPad Prism software v. 10.2. Data were tested for normality with Kolmogorov–Smirnov or Shapiro–Wilk test. Statistical analysis was carried out using one‐way ANOVA followed by Tukey’s or Fisher’s LSD multiple comparisons test. When data did not pass the normality test, the non-parametric Kruskal–Wallis test, with uncorrected Dunn’s test for multiple comparisons, was used. When only two values were compared, the unpaired Student t test was used. A confidence level of 95% was accepted as significant. The number of rats used for each parameter and the statistical procedure used in each case is indicated in the corresponding figure legend.

## 3 Results

### 3.1 Characterization of the size and amount of EV released by CD4^+^ lymphocytes and monocytes from control and hyperammonemic rats

**Figure 2:**
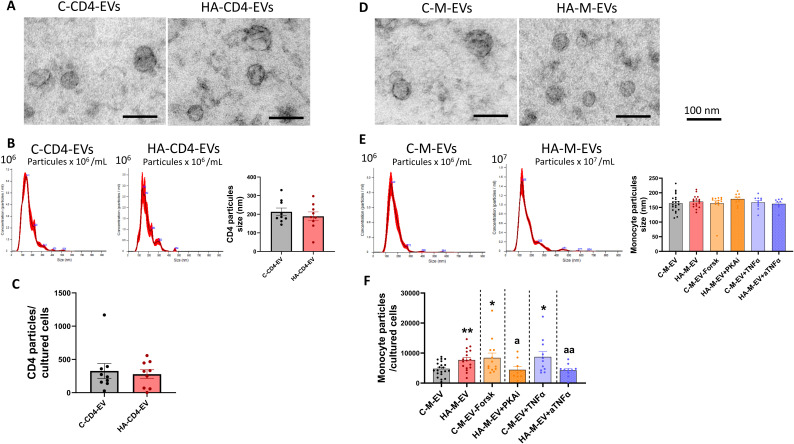
Characterization of the size and amount of EV released by CD4^+^ lymphocytes and monocytes from control and hyperammonemic rats. **(A, D)** TEM images of EV obtained after negative staining reveal vesicles with morphology and dimensions characteristic of small extracellular vesicles (scale bar: 100 µm) **(B, E)** Nanoparticle tracking analysis (NTA) showing a representative size distribution profile of EV, indicating the relative abundance of particles across different size ranges, as well as the average size of particles (n= 9-21). **(C)** The Quantification of the EV released by CD4^+^ lymphocytes per cultured cell assessed by NTA showed no differences between control and hyperammonemic rats (n = 9). **(F)** Quantification of the EV released by monocytes per cultured cell assessed by NTA (n = 9-21). EV release was increased in monocytes from hyperammonemic rats and was normalized by PKAi or anti-TNFα. Forskolin and recombinant TNFα increased EV release by control monocytes, indicating regulation by PKA and TNFα signaling. Values are expressed as percentage of controls. All data are presented as mean ± SEM. Statistical comparisons between control and HA groups were performed using an unpaired t-test. Statistical analysis was determined using four separate one-way ANOVAs, each comparing the two baseline groups (C-M-EV and HA-M-EV) with one of the treatment groups (C-M-EV + Forsk, HA-M-EV + PKAi, C-M-EV + TNFα, or HA-M-EV + aTNFα). The data corresponding to forskolin and recombinant TNFα treatment groups were non-normally distributed and were analyzed using Kruskal–Wallis tests. For the PKAi and anti-TNFα treatment groups, Fisher’s least significant difference (LSD) *post-hoc* tests were applied. Values significantly different from the C-M-EV group are indicated by asterisk (*p<0.05, **p<0.01), and values significantly different from the HA-M-EV group are indicated by a (a=p<0.05, aa=p<0.01).

Electron microscopy images confirmed the isolation of small (<200 nm), round, and concave particles from the culture media of CD4^+^ lymphocytes (**Figure 2A**) and monocytes (**Figure 2D**) from control and hyperammonemic rats.

The NTA shows that for the particles from CD4^+^ cultures, size distribution profiles (**Figure 2B**) and particle concentration (**Figure 2C**) do not differ between CD4^+^ lymphocytes from control and hyperammonemic rats.

For monocytes, the size distribution profiles of the particles are also similar for control and hyperammonemic rats (**Figure 2E**). However, monocytes from hyperammonemic rats release a larger number of particles than control rats (7,702 ± 837 vs. 4,776 ± 534 particles/cultured cell) (**Figure 2F**). Treatment of monocytes from hyperammonemic rats with a PKAi or an anti-TNFα antibody prevented the increase in the release of EV (4,430 ± 1,076, 4,273 ± 662 particles/cultured cell, respectively, **Figure 2F**). Moreover, treatment of control monocytes with forskolin or recombinant TNFα led to a significant increase in EV release (8,403 ± 1,613 particles/cultured cell and 8,750 ± 1,735 particles/cultured cells, respectively, **Figure 2F**). These findings indicate that EV release from monocytes is regulated, at least in part, by PKA and TNFα signaling pathways.

### 3.2 *Ex vivo* incubation with CD4-EV from hyperammonemic rats (HA-CD4-EV) does not induce microglia and astrocyte activation in hippocampal slices from control rats

**Figure 3:**
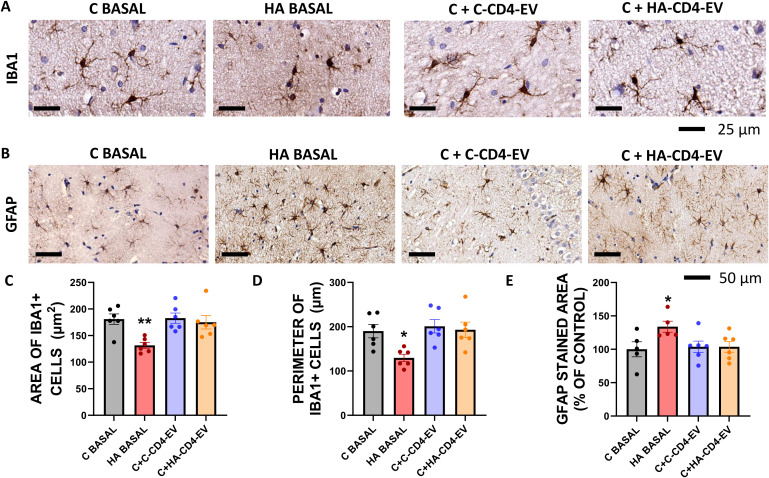
*Ex vivo* incubation with CD4^+^-EV from hyperammonemic rats does not trigger microglial or astrocytic activation in hippocampal slices from control rats. Representative images of immunohistochemistry against Iba1 **(A)** and GFAP **(B)** in hippocampal slices. Quantification of the area **(C)** and perimeter **(D)** of Iba1-positive cells (n = 6) showed a significant reduction in both parameters in hyperammonemic rats, but no significant changes were observed in slices from control rats treated with HA-CD4-EV. The area stained with anti-GFAP **(E)** was increased in hippocampal slices from hyperammonemic rats but remained unchanged in slices from control rats treated with HA-CD4-EV. All data are presented as mean ± SEM. One-way ANOVA followed by Tukey’s (for area and perimeter of iba1+ cells) or by Fisher’s LSD (for GFAP stained area) *post-hoc* tests were performed to compare all groups. Values significantly different from the control group are indicated by asterisk (*p<0.05, **p<0.01). Scale bar = 50 µm, as indicated in the figure.

Hippocampal slices from hyperammonemic rats show microglial activation, as reflected in a reduction in cell area (**Figures 3A, C**) and perimeter (**Figures 3A, D**) of Iba1-stained cells compared with hippocampal slices from control rats. Addition of HA-CD4-EV to hippocampal slices from control rats did not affect the area (**Figures 3A, C**) or perimeter (**Figures 3A, D**) of Iba1-stained cells, indicating that HA-CD4-EV do not induce microglial activation.

HA-CD4-EV also do not induce astrocyte activation (**Figures 3B, E**). The GFAP-stained area was larger in hippocampal slices from hyperammonemic rats compared with control rats, indicating astrocyte activation (**Figures 3B, E**). Addition of HA-CD4-EV to hippocampal slices from control rats did not affect the GFAP-stained area (**Figures 3B, E**), indicating that HA-CD4-EV do not activate astrocytes.

These results show that HA-CD4-EV do not play a significant role in the induction of glial activation and neuroinflammation by PBMC-EV from hyperammonemic rats (HA-PBMC-EV) in hippocampal slices from control rats reported previously (36) and, thus, a different cell type should be responsible for the pathological effects of PBMC-EV.

### 3.3 *Ex vivo* incubation with EV from monocytes of hyperammonemic rats induces microglia and astrocyte activation in hippocampal slices from control rats. The pathological effect of HA-M-EV is regulated by PKA and TNFα

**Figure 4:**
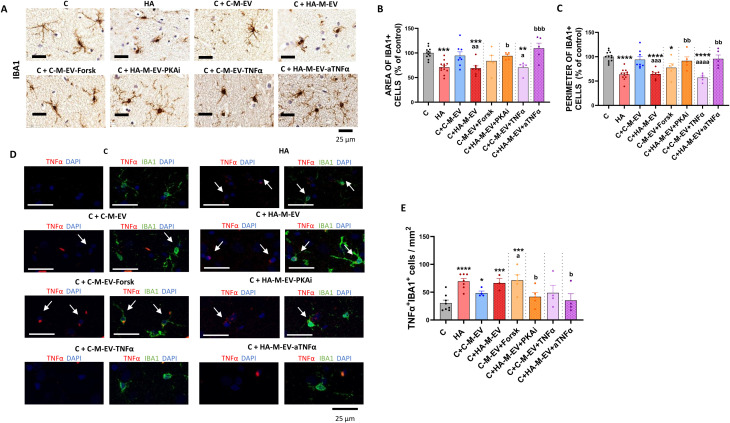
*Ex vivo* incubation with EV from monocytes of hyperammonemic rats (HA-M-EV) induces microglial activation in hippocampal slices from control rats. The pathological effect of EV from monocyte is regulated by PKA and TNFα pathway. **(A)** Representative images of immunohistochemistry against Iba1^+^. Quantification of the area **(B)** and perimeter **(C)** of Iba1^+^ cells (n = 5-12) showed a significant reduction in hippocampal slices from control rats treated with HA-M-EV, but not with HA-M-EV + PKAi or HA-M-EV + aTNFα. C-M-EV + TNFα induced a decrease in both cell area and perimeter, whereas C-M-EV + Forsk reduced only the perimeter of Iba1^+^ cells. **(D)** Representative confocal microscopy images of TNFα^+^ (red) in Iba1^+^ cells (green) by double immunofluorescence of *ex vivo* hippocampal slices. The quantification of Iba1^+^ and TNFα^+^ cells **(E)** showed a significant increase in hippocampal slices from control rats treated with HA-M-EV, which was not observed when EV were derived from HA monocyte cultures treated with either PKAi or anti-TNFα. EV from control monocyte cultures treated with forskolin increased the number of positive cells. All data are presented as mean ± SEM. Statistical analysis was determined using four separate one-way ANOVAs, each comparing the four baseline groups (C, HA, C-M-EV, and HA-M-EV) with one of the treatment groups (C-M-EV + Forsk, HA-M-EV + PKAi, C-M-EV + TNFα, or HA-M-EV + aTNFα). One-way ANOVA followed by Tukey’s (for area and perimeter of iba1^+^ cells) or by Fisher’s LSD (for number of Iba1- and TNFα-positive cells/mm^2^) *post-hoc* tests were performed to compare all groups. Values significantly different from the C group are indicated by asterisk (*p<0.05, **p<0.01, ***p<0.001, ****p<0.0001), values significantly different from the C-M-EV group are indicated by a (a=p<0.05, aa=p<0.01, aaa=p<0.001, aaaa=p<0.0001), and values significantly different from the HA-M-EV group are indicated by b (b=p<0.05, bb=p<0.01, bbb=p<0.001). Scale bar = 25 µm, as indicated in the figure.

*Ex vivo* treatment of hippocampal slices from control rats with HA-M-EV induced microglial activation similar to that observed in hippocampal slices from hyperammonemic rats. Both the cell area (**Figures 4A, B**) and perimeter (**Figures 4A, C**) were reduced when slices were treated with HA-M-EV, but not with C-M-EV from control rats.

Addition to hippocampal slices from control rats of EV from HA-monocytes cultured in the presence of an inhibitor of PKA (HA-M-EV+PKAi) or of anti-TNFα (HA-M-EV+a TNFα) did not affect the area (**Figures 4A, B**) or perimeter (**Figures 4A, C**) of Iba1-stained cells, indicating that the EV generated by these HA monocytes have lost the pathological effects. This supports that both PKA activation and TNFα in the HA monocytes contribute to the generation of pathological EV.

To assess if PKA activation and/or TNFα are enough to induce the generation of pathological EV, we treated monocytes from control rats with forskolin, an activator of adenylate cyclase that increases cAMP and PKA activation, or with recombinant TNFα.

EV from control monocytes treated with forskolin (C-M-EV+Forsk) or recombinant TNFα (C-M-EV+TNFα) induce microglial activation, reducing the perimeter (**Figures 4A, C**) of Iba1-stained cells when adding *ex vivo* to hippocampal slices and the area only in the case of C-M-EV+TNFα treatment (**Figures 4A, B**). This indicates that monocytes from control rats also release pathological EV if PKA activation or TNFα levels are increased.

As an additional way to analyze pro-inflammatory activated microglia, we also measured the content of TNFα into microglial cells by double immunofluorescence with anti-Iba1 and anti-TNFα in the hippocampal slices.

Hippocampal slices from hyperammonemic rats exhibit pro-inflammatory microglia, with an increased number of Iba1^+^ cells co-expressing TNFα compared with control rats (**Figures 4D, E**). Addition of HA-M-EV to hippocampal slices from control rats also induced pro-inflammatory activation of microglia, with increased expression of TNFα (**Figures 4D, E**). The increase in TNFα^+^ in Iba^+^ cells was not induced by EV from HA monocytes cultured in the presence of an inhibitor of PKA (HA-M-EV+PKAi) or of anti-TNFα (HA-M-EV+a TNFα) and was also induced by EV from control monocytes treated with forskolin (C-M-EV+Forsk) (**Figures 4D, E**).

**Figure 5:**
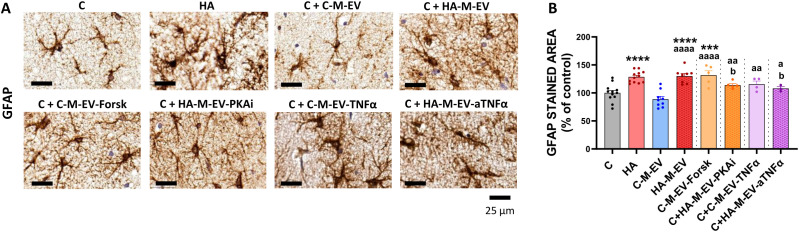
*Ex vivo* incubation with EV from monocytes of hyperammonemic rats (HA-M-EV) induces astrocyte activation in hippocampal slices from control rats. The pathological effect of EV from monocyte is regulated by PKA and TNFα pathway. **(A)** Representative images of immunohistochemistry against GFAP. The quantification of the percentage of the area stained by anti-GFAP **(B)** showed an increase in hippocampal slices from control rats treated with HA-M-EV. EV from HA monocyte cultures treated with PKAi or anti-TNFα induced a significantly smaller increase in the GFAP-stained area compared with HA-M-EV. EV from control monocyte cultures treated with forskolin or recombinant TNFα induced an increase in the percentage of GFAP-stained area. All data are presented as mean ± SEM. Statistical analysis was determined using four separate one-way ANOVAs, each comparing the four baseline groups (C, HA, C-M-EV, and HA-M-EV) with one of the treatment groups (C-M-EV+ Forsk, HA-M-EV + PKAi, C-M-EV + TNFα, or HA-M-EV + aTNFα). One-way ANOVA followed by Fisher’s *post-hoc* tests were performed to compare all groups. Values significantly different from the C group are indicated by asterisk (***p<0.001, ****p<0.0001), values significantly different from the C-M-EV group are indicated by a (a=p<0.05, aa=p<0.01, aaa=p<0.001), and values significantly different from the HA-M-EV group are indicated by b (b=p<0.05). Scale bar = 25 µm, as indicated in the figure.

Astrocyte activation was analyzed by quantifying the percentage of the area stained by anti-GFAP. The GFAP-stained area was increased in hippocampal slices from hyperammonemic rats and in hippocampal slices from control rats treated with HA-M-EV (**Figures 5A, B**). The increase in GFAP induced by HA-M-EV was not induced when monocytes were treated with an inhibitor of PKA (HA-M-EV+PKAi) or anti-TNFα (HA-M-EV+a TNFα). Astrocyte activation was also observed in response to EV from control monocytes treated with forskolin (C-M-EV+Forsk) or TNFα (C-M-EV+TNFα) (**Figures 5A, B**).

These results show that, in hyperammonemia, EV released by monocytes (HA-M-EV) but not those released by CD4^+^ lymphocytes (HA-CD4-EV) induce glial activation in control rats similar to that observed in hippocampal slices from hyperammonemic rats and would play a significant role in the pathological effects on neuroinflammation in the hippocampus in hyperammonemic rats. Interestingly, the induction of microglia and astrocyte activation by HA-M-EV is prevented if the HA monocytes are treated with an inhibitor of PKA or with anti-TNFα.

### 3.4 EV from monocytes of hyperammonemic rats activate the TNFα–TNFR1–S1PR2–IL-1β–BDNF–TrkB pathway and alter the membrane expression of NMDA and AMPA receptor subunits in hippocampal slices from control rats

In hyperammonemic rats, neuroinflammation in the hippocampus alters the membrane expression of NMDA and AMPA receptors leading to cognitive impairment. This is mediated by enhanced activation of the TNFα–TNFR1–S1PR2–IL-1β–BDNF–TrkB pathway and the IL-1β–NR2B–GluA1–GluA2 pathway (26, 42, 48). EV from plasma or from PBMCs of hyperammonemic rats enhance activation of these pathways, leading to cognitive impairment (26, 36).

**Figure 6:**
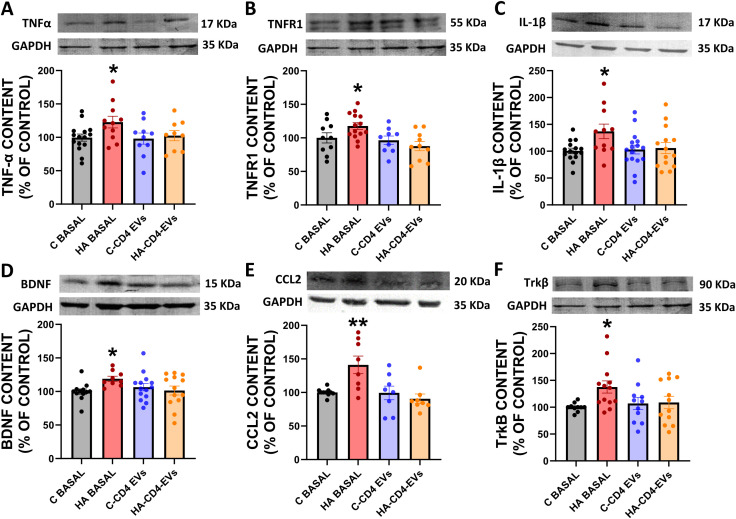
*Ex vivo* incubation with EV from CD4^+^ of hyperammonemic rats (HA-M-EV) does not induce TNFα–TNFR1–S1PR2–IL-1β–BDNF pathway activation in hippocampal slices from control rats. Protein content of **(A)** TNFα (n= 9-15), **(B)** TNFR1 (n= 9-14), **(C)** IL-1β (n= 11-15), **(D)** BDNF (n= 9-14), **(E)** CCL2 (n= 8), and **(F)** TrkB (n= 10-13) in hippocampal slices, assessed by Western blot. Representative images of the blots of each protein are shown. All data are presented as mean ± SEM. One-way ANOVA followed by Tukey’s (TNFα, TNFR1, IL-1β, BDNF, TrkB) *post-hoc* tests were performed to compare all groups. The data corresponding to CCL2 content were non-normally distributed and were analyzed using Kruskal–Wallis tests. Values significantly different from control group are indicated by asterisk (*p<0.05, **p<0.01).

We therefore analyzed the effects of addition of HA-CD4-EV or HA-M-EV to hippocampal slices from control rats. Addition of HA-CD4-EV to hippocampal slices from control rats did not affect the levels of TNFα, TNFR1, IL-1β, CCL2, BDNF, or TrkB levels (**Figures 6A–F**), in agreement with the lack of effect on microglia and astrocyte activation shown in **Figure 3**.

**Figure 7:**
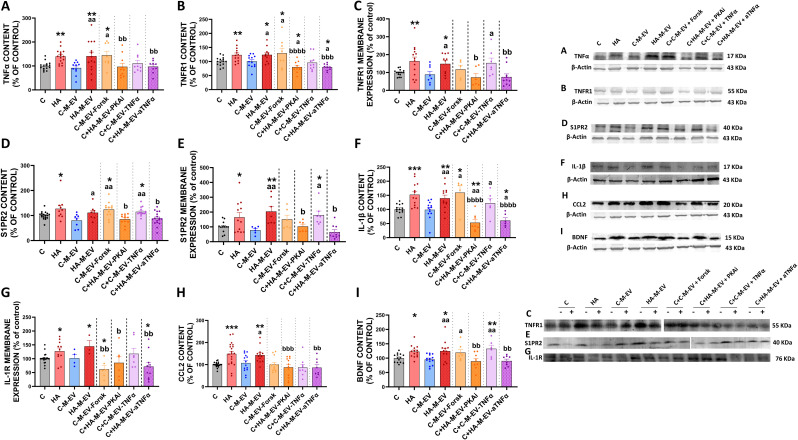
*Ex vivo* incubation with EV from monocytes of hyperammonemic rats (HA-M-EV) induces TNFα–TNFR1–S1PR2–IL-1β–BDNF pathway activation in hippocampal slices from control rats. Protein content of **(A)** TNFα (n= 8-21), **(B)** TNFR1 (n= 8-15), **(D)** S1PR2 (n= 8-18), **(F)** IL-1β (n= 5-14), **(H)** CCL2 (n= 8-21), and **(I)** BDNF (n= 6-14) and membrane expression of **(C)** TNFR1 (n= 5-15), **(E)** S1PR2 (n= 6-12), and **(G)** IL1R (n= 3-20) in hippocampal slices, assessed by Western blot. For the analysis of membrane expression, the sections were incubated in the presence (+) or absence (−) of the cross-linker BS3. Samples in the absence of BS3 represent the total amount of each protein, whereas samples incubated in the presence of BS3 represent the non-membrane fraction of each protein. Representative images of the blots of each protein are shown. All data are presented as mean ± SEM. Statistical analysis was determined using four separate one-way ANOVAs, each comparing the four baseline groups (C, HA, C-M-EV, and HA-M-EV) with one of the treatment groups (C-M-EV + Forsk, HA-M-EV + PKAi, C-M-EV + TNFα, or HA-M-EV + aTNFα). One-way ANOVA followed by Fisher’s *post-hoc* tests were performed to compare all groups. Values significantly different from the C group are indicated by asterisk (*p<0.05, **p<0.01, ***p<0.001), values significantly different from the C-M-EV group are indicated by a (a=p<0.05, aa=p<0.01), and values significantly different from the HA-M-EV group are indicated by b (b=p<0.05, bb=p<0.01, bbb=p<0.001, bbbb=p<0.0001).

**Figure 8:**
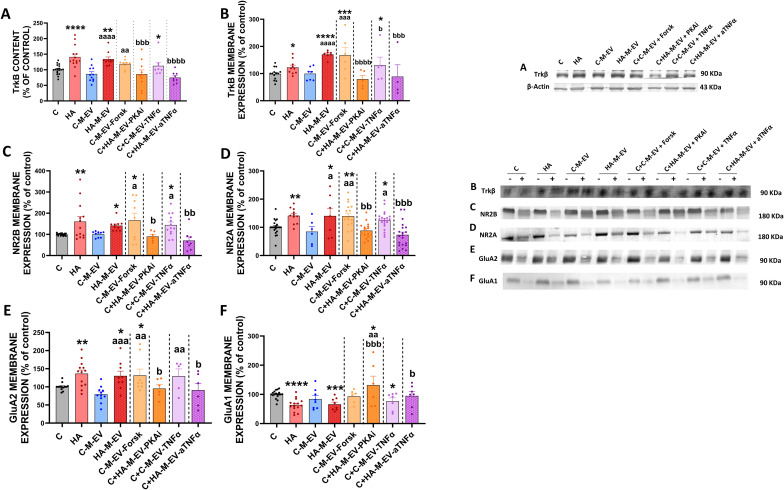
*Ex vivo* incubation with EV from monocytes of hyperammonemic rats (HA-M-EV) induces TrkB–NR2B–GluA1–GluA2 pathway activation in hippocampal slices from control rats. Protein content of **(A)** TrkB (n= 8-18) and membrane expression of **(B)** TrkB (n= 4-14), **(C)** NR2B (n= 7-16), **(D)** NR2A (n= 6-22), **(E)** GluA2 (n= 5-13), and **(F)** GluA1 (n= 6-18) in hippocampal slices, assessed by Western blot. For the analysis of membrane expression, the sections were incubated in the presence (+) or absence (−) of the cross-linker BS3. Samples in the absence of BS3 represent the total amount of each protein, whereas samples incubated in the presence of BS3 represent the non-membrane fraction of each protein. Representative images of the blots of each protein are shown. All data are presented as mean ± SEM. Statistical analysis was determined using four separate one-way ANOVAs, each comparing the four baseline groups (C, HA, C-M-EV, and HA-M-EV) with one of the treatment groups (C-M-EV + Forsk, HA-M-EV + PKAi, C-M-EV + TNFα, or HA-M-EV + aTNFα). One-way ANOVA followed by Fisher’s *post-hoc* tests were performed to compare all groups. Values significantly different from the C group are indicated by asterisk (*p<0.05, **p<0.01, ***p<0.001, ****p<0.0001), values significantly different from the C-M-EV group are indicated by a (a=p<0.05, aa=p<0.01, aaa=p<0.001, aaaa=p<0.0001), and values significantly different from HA-M-EV group are indicated by b (b=p<0.05, bb=p<0.01, bbb=p<0.001, bbbb=p<0.0001).

In contrast, addition of HA-M-EV to slices from control rats induce the same alterations than hyperammonemia per se on the TNFα–TNFR1–S1PR2–IL-1β–BDNF–TrkB pathway and the IL-1β–NR2B–GluA1–GluA2 pathway. HA-M-EV increase TNFα content (**Figure 7A**), the content (**Figure 7B**) and membrane expression of TNFR1 (**Figure 7C**), the content (**Figure 7D**) and membrane expression of S1PR2 (**Figure 7E**), IL-1β levels (**Figure 7F**), membrane expression of its receptor IL-1R (**Figure 7G**), the content of CCL2 (**Figure 7H**) and BDNF (**Figure 7I**), and the content (**Figure 8A**) and membrane expression (**Figure 8B**) of TrkB and of the NR2B subunit of NMDA receptors (**Figure 8C**) and of the GluA2 subunit of AMPA receptors (**Figure 8D**) while reducing the membrane expression of GluA1 (8E).

All these changes induced by HA-M-EV in the TNFα–TNFR1–S1PR2–IL-1β–BDNF–TrkB pathway and the IL-1β–NR2B–GluA1–GluA2 pathway were not induced when monocytes were treated with an inhibitor of PKA (HA-M-EV+PKAi) or with anti-TNFα (HA-M-EV+a TNFα) (**Figures 7**, **8**).

EV from monocytes of control rats (C-M-EV) did not affect the TNFα–TNFR1–S1PR2–IL-1β–BDNF–TrkB pathway or the IL-1β–NR2B–GluA1–GluA2 pathway (**Figures 7**, **8**).

However, the EV from control monocytes treated with forskolin (C-M-EV+Forsk) induced most of the changes in these pathways induced by hyperammonemia, except for the changes in membrane expression of TNFR1, S1PR2, and GluA1 and the increase in IL-1 receptor and CCL2 (**Figures 7**, **8**; **Table 1**).

**Table 1:** Effects of the different treatments on glial activation and on the steps of the TNFα–TNFR1–S1PR2–IL-1β–BDNF–TrkB pathway and membrane expression of NMDA and AMPA receptor subunits.

Protein/cell type	Parameter	HA-M-EV	HA-M-EV-anti-TNFa	HA-M-EV-PKAi	C-M-EV-TNFa	C-M-EV-Fosk
TNFa	Content				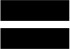	
TNFR1	Content				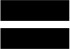	
TNFR1	Membrane expression					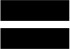
S1PR2	Content					
S1PR2	Membrane expression					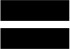
IL1B	Content					
IL1R	Membrane expression				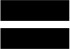	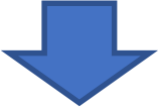
CCL2	Content				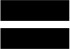	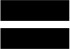
BDNF	Content					
TrkB	Content					
TrkB	Membrane expression					
NR2B	Membrane expression					
NR2A	Membrane expression					
GluA2	Membrane expression					
GluA1	Membrane expression	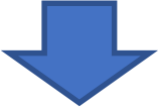	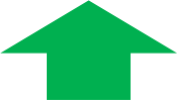	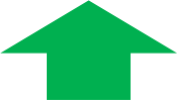	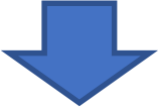	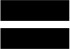
Microglia	Activation					
Microglia	TNFα expression				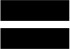	
Astrocytes	Activation					

Blue arrows indicate changes statistically significant versus control slices. Green arrows indicate reversal of the effects induced by HA-M-EV.

EV from control monocytes treated with TNFα (C-M-EV+TNFα) also induced some of the effects induced by hyperammonemia on these pathways, except for the increase of TNFα in microglia (**Figures 4D, E**) and in the whole hippocampus (**Figure 7A**) and the increases in TNFR1, IL-1 receptor, and CCL2 (**Figures 7**, **8**; **Table 1**).

These data show that adding HA-M-EV to hippocampal slices from control rats enhances activation of the TNFα–TNFR1–S1PR2–IL-1β–BDNF–TrkB pathway and alters the membrane expression of NMDA and AMPA receptors in a manner similar to that observed *in vivo* in hyperammonemia. Moreover, the production of pathological EV by HA-M-EV is regulated by PKA and TNFα.

### 3.5 The contents of TNFR1 and of TNFα are increased in the EV released by monocytes of hyperammonemic rats and are normalized by inhibiting PKA or blocking TNFα

We have previously shown that the pathological effects of EV from plasma or PBMC of hyperammonemic rats are mediated by the TNFα-TNFR1 system (23, 36). We therefore analyzed the content of these proteins and of EV markers in EV from CD4^+^ and monocytes of hyperammonemic and control rats.

**Figure 9:**
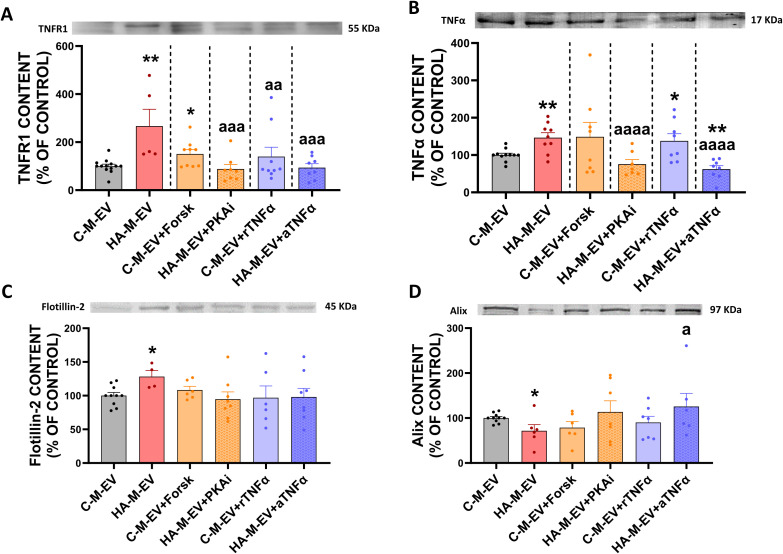
Contents of TNFR1 and TNFα are increased in the EV released by monocytes of hyperammonemic rats and are normalized by inhibiting PKA or blocking TNFα. Protein content of **(A)** TNFR1 (n= 5-13), **(B)** TNFα (n= 7-11), **(C)** Flotillin-2 (n= 4-10), and **(D)** Alix (n= 6-10), in EV from monocyte cells, assessed by Western blot. Representative images of the blots of each protein are shown. Values are expressed as percentage of controls. All data are presented as mean ± SEM. Statistical comparisons between C-M-EV and HA-M-EV groups were performed using an unpaired t-test. Statistical analysis was determined using four separate one-way ANOVAs, each comparing the two baseline groups (C-M-EV and HA-M-EV) with one of the treatment groups (C-M-EV + Forsk, HA-M-EV + PKAi, C-M-EV + TNFα, or HA-M-EV + aTNFα). The data corresponding to TNFR1 content were non-normally distributed and were analyzed using Kruskal–Wallis tests. For TNFα, Flotillin-2, and Alix contents, Fisher’s least significant difference (LSD) *post-hoc* tests were applied. Values significantly different from the C-M-EV group are indicated by asterisk (*p<0.05, **p<0.01), and values significantly different from HA-M-EV group are indicated by a (a=p<0.05, aa=p<0.01, aaa=p<0.01).

We observed that TNFR1 and TNF-α levels are not increased in HA-CD4-EV compared with C-CD4-EV (data not shown). However, HA-M-EV show increased content of TNFR1 (**Figure 9A**), TNFα (**Figure 9B**), and flotillin 2 (**Figure 9C**) and reduced content of Alix (**Figure 9D**). All these changes were prevented by treating the HA monocytes with an inhibitor of PKA or with anti-TNFα (**Figure 9**).

Treatment of control monocytes with forskolin increased the EV content of TNFR1 (**Figure 9A**), whereas its effect on TNFα content was not statistically significant (**Figure 9B**), and it did not affect flotillin-2 or Alix. In contrast, treatment with TNFα significantly increased the EV content of TNFα (**Figure 9B**) but did not modify TNFR1, flotillin-2, or Alix levels (**Figure 9**). As an additional control, we confirmed the presence of the exosome marker CD9 in EVs from monocytes, supporting the presence of small EVs/exosomes in our preparations (data not shown).

### 3.6 The contents of cAMP and of TNFR1, TNFα, glutaminase, and CCR2 are increased in monocytes of hyperammonemic rats and are normalized by inhibiting PKA or blocking TNFα

**Figure 10:**
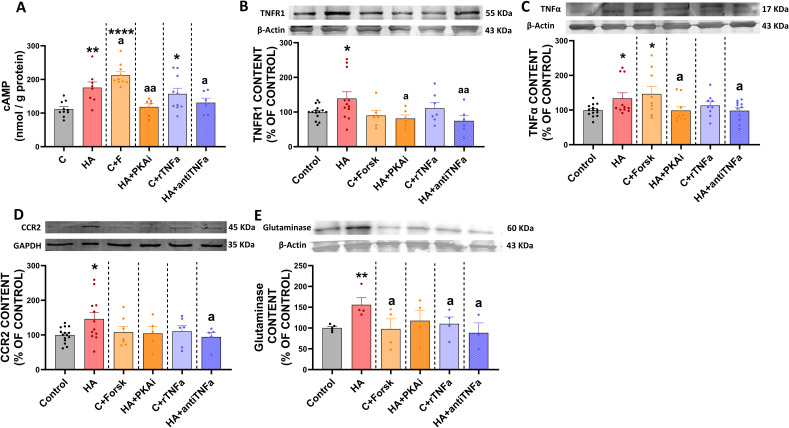
Contents of cAMP and of TNFR1, TNFα, glutaminase, and CCR2 are increased in monocytes of hyperammonemic rats and are normalized by inhibiting PKA or blocking TNFα. **(A)** cAMP levels, as assessed by LC-MS, and protein content of **(B)** TNFR1 (n= 6-14) **(C)** TNFα (n= 9-14), **(D)** CCR2 (n= 5-15), and **(E)** glutaminase (n= 3-5) in monocyte cells, assessed by Western blot, expressed as percentage of the control group. Representative images of the blots of each protein are shown. All data are presented as mean ± SEM. Statistical comparisons between the C-M-EV and HA-M-EV groups were performed using an unpaired t-test. Statistical analysis was determined using four separate one-way ANOVAs, each comparing the two baseline groups (C, HA) with one of the treatment groups (C-M-EV + Forsk, HA-M-EV + PKAi, C-M-EV + TNFα, or HA-M-EV + aTNFα). The data corresponding to TNFR1 content were non-normally distributed and were analyzed using Kruskal–Wallis tests. For TNFα, CCR2, and glutaminase contents, Fisher’s least significant difference (LSD) *post-hoc* tests were applied. Values significantly different from the C group are indicated by asterisk (*p<0.05, **p<0.01,****p<0.0001), and values significantly different from HA group are indicated by a (a=p<0.05, aa=p<0.01).

The above data suggest that hyperammonemia enhances the cAMP-PKA system in monocytes. We therefore analyzed the cAMP levels, which were increased in monocytes from hyperammonemic rats (**Figure 10A**) and are normalized by inhibiting PKA or blocking TNFα with anti-TNFα. cAMP levels are also increased in control monocytes by treatment with forskolin or TNFα (**Figure 10A**).

As TNFR1 and TNFα are increased in HA-M-EV, we analyzed whether these proteins are also increased in the monocytes themselves. TNFR1 (**Figure 10B**) and TNFα (**Figure 10C**), as well as glutaminase (**Figure 10D**) and CCR2 (**Figure 10E**), were increased in monocytes from hyperammonemic rats. All these increases are reversed by inhibiting PKA or blocking TNFα with anti-TNFα (**Figures 10B–E**). Treatment of control monocytes with forskolin increased TNFα, but not the other proteins. Treatment of control monocytes with recombinant TNFα did not alter the expression of either protein (**Figures 10B–E**). The lack of an intracellular increase in TNFα may reflect its preferential secretion within the EV cargo (**Figure 9B**).

### 3.7 Hyperammonemia induces lysosomal dysfunction and reduces LC3 content in monocytes

It has been proposed that lysosomal dysfunction is responsible for the increased secretion of EV in different situations, including astrocytes overexpressing α-synuclein (38).

**Figure 11:**
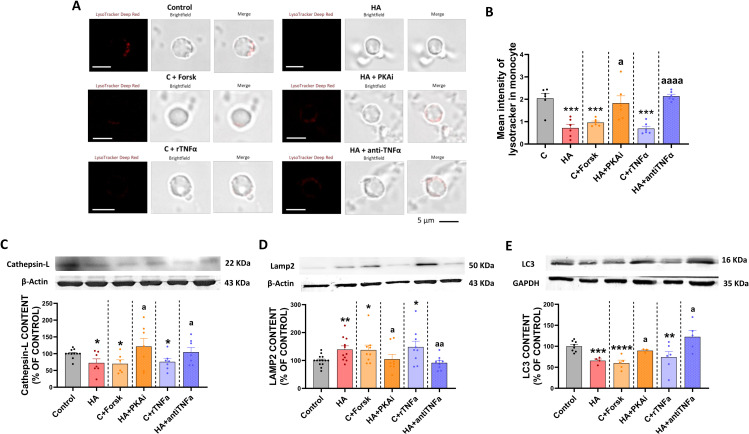
Monocytes from hyperammonemic rats exhibit lysosomal dysfunction-related markers, which are regulated by PKA and TNFα pathways. **(A)** Representative confocal microscopy images of LysoTracker Red dye (red) in monocyte cells (bright field). **(B)** Quantification of the mean fluorescence intensity of LysoTracker Red dye in monocyte cells (n= 6). Protein content of **(C)** Cathepsin-L (n=), **(D)** Lamp2 (n= 6-11), and **(E)** LC3 (n= 3-8) in monocyte cells, assessed by Western blot, expressed as percentage of the control group. Representative images of the blots of each protein are shown. All data are presented as mean ± SEM. Statistical comparisons between C-M-EV and HA-M-EV groups were performed using an unpaired t-test. Statistical analysis was determined using four separate one-way ANOVAs, each comparing the two baseline groups (C, HA) with one of the treatment groups (C-M-EV + Forsk, HA-M-EV + PKAi, C-M-EV + TNFα, or HA-M-EV + aTNFα). One-way ANOVA followed by Fisher’s (for Cathepsin-L, Lamp2, LC3) or by Tukey’s (for lysotracker) *post-hoc* tests were performed to compare all groups. Values significantly different from the C group are indicated by asterisk (*p<0.05, **p<0.01, ***p<0.001, ****p<0.0001), and values significantly different from HA group are indicated by a (a=p<0.05, aa=p<0.01, aaaa=p<0.0001). Scale bar = 5 µm, as indicated in the figure.

We therefore analyzed lysosomal function in monocytes. We found that hyperammonemia induces lysosomal dysfunction, with reduced LysoTracker staining (**Figures 11A, B**) and cathepsin L levels (**Figure 11C**) and increased LAMP2 content (**Figure 11D**). All these changes are reversed by inhibiting PKA or blocking TNFα with anti-TNFα (**Figures 11A–D**). Treatment of control monocytes with forskolin or TNFα also induced lysosomal dysfunction, with reduced LysoTracker staining (**Figures 11A, B**) and cathepsin L levels (**Figure 11C**) and increased LAMP2 content (**Figure 11D**).

We also analyzed the content of LC3, a main modulator of autophagy, in monocytes. Hyperammonemia reduces the LC3 content in monocytes, and this effect is reversed by inhibiting PKA or blocking TNFα with anti-TNFα (**Figure 11E**). Treatment of control monocytes with forskolin or TNFα also reduced LC3 content (**Figure 11E**).

## 4 Discussion

We have previously shown that injection to normal rats of EV from plasma or PBMCs from hyperammonemic rats induces cognitive impairment, which is mediated by induction of neuroinflammation and alterations in glutamatergic neurotransmission in the hippocampus. The mechanisms by which these EV alter neuroinflammation and neurotransmission were identified in detail in *ex vivo* experiments by adding the EV from hyperammonemic rats to hippocampal slices from control rats. The underlying mechanisms were identical for EV from plasma or from PBMCs of hyperammonemic rats (26, 36).

A relevant pending question was which cell type within the PBMC produces the pathological EV in hyperammonemic rats. We show here that EV from monocytes but not from CD4^+^ lymphocytes from hyperammonemic rats induce the pathological effects. When added to hippocampal slices from control rats, the EV from HA monocytes induce identical effects to those induced by EV from plasma or PBMCs from hyperammonemic rats. EV from monocytes induce microglial activation, as indicated by the reduced area and perimeter of Iba1-stained microglia cells and by the increased TNFα content in microglia. EV from monocytes also activate astrocytes, as indicated by the increased area covered by anti-GFAP staining.

This glial activation was not induced by addition of EV released by CD4^+^ lymphocytes from hyperammonemic rats, indicating that the EV from monocytes are pathological, whereas those from CD4^+^ lymphocytes are not.

EV from monocytes (but not from CD4^+^ lymphocytes) also enhance the activation of the TNFα–TNFR1–S1PR2–IL-1β–IL-1 receptor–CCL2–BDNF–TrkB pathway and alter the membrane expression of the NR2B subunit of NMDA receptors and GluA1 and GluA2 subunits of AMPA receptors in hippocampal slices from control rats. These effects are identical to those induced by EV from plasma or from PBMCs of hyperammonemic rats (26, 36). Our previous studies have shown that the TNFα–TNFR1–S1PR2–IL-1β–BDNF–TrkB pathway mediates glial activation in the hippocampus and cerebellum of hyperammonemic rats. This pathway has been confirmed in previous studies of our laboratory using different inhibitors. In (14), the *in vivo* treatment with infliximab, an anti–TNFα antibody, prevented microglial and astrocyte activation in the hippocampus induced by hyperammonemia. Infliximab treatment also prevented changes in membrane expression of GluA1 and GluA2 subunits of AMPA receptors and NR2A and NR2B subunits of NMDA receptors and normalized cognitive deficits.

In (49), cerebellar slices from hyperammonemic rats were incubated with R7050, a TNFR signaling inhibitor that blocks the formation of the TNFR1–TRADD/RIP1/TRAF complex. Remarkably, R7050 strongly reduced TNFα expression in Purkinje neurons, microglia, and astrocytes, confirming that TNFα upregulation induced by hyperammonemia is mediated by TNFR1 activation and not by other TNF receptors, such as TNFR2. In (48), it was found that the SP1PR2-CCL2-CCR2-BDNF-TrkB pathway is also involved in the inflammatory process in the hippocampus of hyperammonemic rats. Blocking S1PR2 with JTE-013 *in vivo* and *ex vivo* reversed microglial and astrocyte activation and the increase in IL-1β. JTE-013 also restored the altered membrane expression of AMPA and NMDA receptors and improved learning and spatial memory in hyperammonemic rats. Microglial activation was also reversed by blocking the IL-1 receptor or CCR2, supporting a key role for this pathway Activation of this pathway has also been demonstrated in the cerebellum of hyperammonemic rats (50).

These data indicate that monocytes are the source of the pathological EVs found in the plasma of hyperammonemic rats, which drive neuroinflammation, alter neurotransmission, and impair cognitive function. Importantly, TNFR1 activation by TNFα contained in these EVs represents a critical upstream step in hyperammonemia-induced glial activation.

A key role for monocytes in the induction of neuroinflammation by peripheral inflammation was also reported by (51). They showed that injection of lipopolysaccharide (LPS) to rats induces peripheral inflammation and neuroinflammation with microglial activation and increased levels of IL-1β and TNFα in substantia nigra. Depletion of monocytes by injection of clodronate liposomes strongly reduced LPS-induced microglial activation and the increase in IL-1β and TNFα.

We have also shown that hyperammonemia increases the amount of EV in plasma and in the cultures of PBMCs from hyperammonemic rats (26, 36). We show here that monocytes (but not CD4^+^ lymphocytes) from hyperammonemic rats also release a larger amount of EV than the monocytes from control rats.

**Figure 12:**
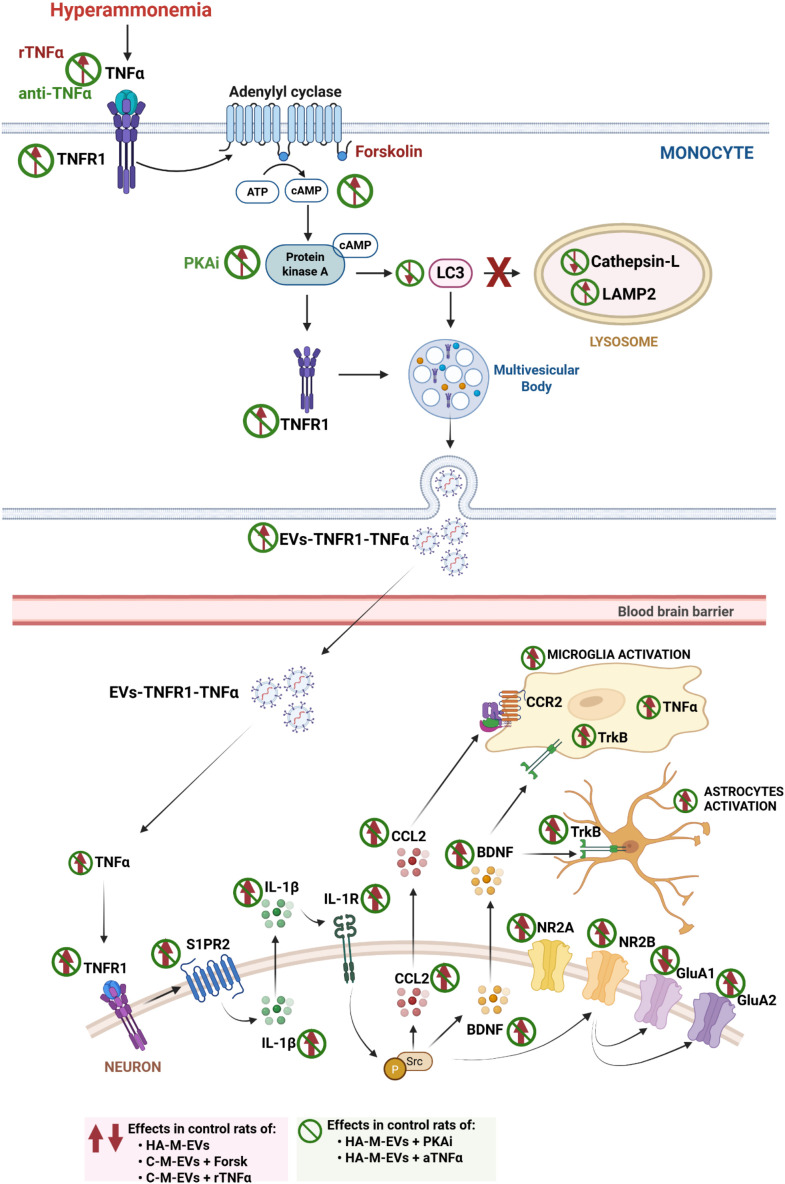
Schematic representation of the proposed mechanism by which (1) hyperammonemia induces the formation of pathological EV containing TNFR1-TNFα in monocytes, (2) how TNFα and PKA modulate this process, and (3) how these EV induce pathological effects in the hippocampus. Hyperammonemia increases TNF α and activation of its receptor TNFR1 in monocytes, leading to activation of adenylate cyclase and increased cAMP levels and PKA activation. PKA alters both LC3, leading to increased lysosomal pH and impaired expression of lysosomal proteins such as cathepsin-L and LAMP2. This results in lysosomal dysfunction and altered autophagic flux enhances the release from multivesicular bodies (MVBs) of EV containing increased levels of TNFR1 and TNFα. These pathological HA-M-EV enhance activation of the TNFα–TNFR1–S1PR2–IL-1β–BDNF pathway and the TrkB–NR2B–GluA1–GluA2 pathway in hippocampal slices from control rats, inducing neuroinflammation and altered glutamatergic neurotransmission. All these effects are reversed by blocking TNFα with anti-TNFα or inhibiting PKA with H69 in the cultures of monocytes from hyperammonemic rats (green symbols). Activation of TNFR1 with recombinant TNFα (rTNFα) or of adenylate cyclase with forskolin in monocyte cultures from control rats induce essentially the same effects (red arrows). This figure was created using Biorender.com.

Here, we also identify the key mechanisms underlying the increase in EV production and the formation of pathological EV in HA monocytes. The results are summarized in **Figure 12**. We show that increased levels of TNFα in monocytes from hyperammonemic rats lead to increased levels of cAMP and activation of PKA which induces an increase in the release of EV by inducing lysosomal dysfunction and a decrease in LC3 as well as an increase in the content of TNFR1 and TNFα in the EV which would mediate their pathological effects in the hippocampus.

There are several reports showing that TNFα increases the release of EV in different cell types including mesenchymal stromal cells (52, 53) and mouse astrocytes (54). Synovial fibroblasts from individuals with rheumatoid arthritis (RASF) contain a membrane-bound form TNFα. The RASF-derived EV increase the release of EV from the L929 cell. Blocking TNFα reduces EV production (55). Similar results have been observed in the microglial cell line BV2. Activation of BV2 microglial cells with lipopolysaccharide increases the EV levels of TNFα as well as the number of EV released (56). Both the increase in the number of EV and in the content of TNFα in the EV were reduced by addition of etanercept, which acts as a soluble TNFα receptor and blocks TNFα (56). TNFα also triggers the release of EV containing TNFR1, which can modulate TNFα responses of the parental cells (57).

All these reports support that TNFα increases the release of EV and the TNFR1 and TNFα content of the EV in different cell types, as we observed in EVs from hyperammonemic monocytes. We also show that blocking TNFα with an anti-TNFα reduces the amount of EV released. Moreover, adding recombinant TNFα to the cultures of monocytes from normal rats also induces an increase in the amount of EV released. These data support that the increased levels of TNFα and the activation of its receptor are responsible for the enhanced production of EV in monocytes from hyperammonemic rats.

Moreover, we show that adding anti-TNFα to the primary cultures of monocytes from hyperammonemic rats reduces the levels of TNFR1 and TNFα in the EVs, and these EVs no longer induce glial activation, neuroinflammation, activation of the TNFα–TNFR1–S1PR2–IL-1β–IL-1 receptor–CCL2–BDNF–TrkB pathway, or alterations in the membrane expression of NMDA or AMPA receptors when added to hippocampal slices from control rats.

This indicates that TNFα triggers the production of pathological EV in monocytes from hyperammonemic rats. This is further supported by the fact that adding recombinant TNFα to monocytes from control rats induce the formation of pathological EV that induce glial activation, neuroinflammation, and altered glutamatergic neurotransmission.

We then aimed to identify the mechanisms by which TNFα induces these pathological effects. We have shown that EV from hyperammonemic rats carry increased levels of TNFα and of its receptor TNFR1, which play a key role in the pathological effects of the EV (26, 36).

Islam et al. (37) showed that cAMP induces the release of TNFR1 in exosome-like vesicles via a PKA-dependent mechanism. We therefore assessed if enhanced activation of PKA in HA monocytes mediates the release of pathological EV. We show that hyperammonemia increases the levels of cAMP in monocytes and that adding an inhibitor of PKA to the monocyte cultures from hyperammonemic rats also prevents the formation of pathological EV. The EV released under these conditions no longer induce pathological effects in hippocampal slices from control rats. Moreover, the inhibitor of PKA also reverses the increase in the number of EV released by monocytes from hyperammonemic rats.

In addition, increasing cAMP and PKA activity in monocytes from normal rats by addition of forskolin also induces the production of pathological EV that induce neuroinflammation and altered neurotransmission and increase the number of EV released.

These data indicate that cAMP-PKA are also involved in the production of pathological EV and in the increased number of EV.

We show that the increase in cAMP in monocytes from hyperammonemic rats is reversed by addition of anti-TNFα to the culture, indicating that TNFα induces the increase in cAMP and PKA activation, likely by activating an adenylate cyclase, which has been reported in different cell types. For example, TNFα activates Ca-dependent adenylyl cyclase and increases cAMP in neutrophils (58–63). TNFα increases cAMP and activates the cAMP/PKA signaling pathway in synovial fibroblasts (SFs) from rheumatoid arthritis (RA) patients (64). Klukovic et al. (65) propose that TNFα increases cAMP in the uterus of pregnant rats.

cAMP increases the activity of PKA which triggers the release of pathological EV containing increased levels of TNFR1 and TNFα. Inhibiting PKA eliminates the pathological effects of the EV by reducing their TNFR1 and TNFα content. Moreover, inhibiting PKA also eliminates the increase in the amount of EV released by the monocytes, indicating a role for the increased PKA activity in the enhanced release of EV. The PKA-induced increase of EV release would be due to impairment of lysosomal function and autophagy. It has been shown that cAMP and PKA activity modulates autophagy, lysosomal function, and pH and cathepsin D levels. PKA inhibits autophagy by phosphorylating Atg1, Atg13, and Atg12 (66–68). Increased cAMP/PKA signaling results in decreased content of autophagosomes in luteal cells (69) and also inhibits autophagy in fasting (70). Forskolin increases cAMP and lysosomal membrane permeabilization and modulates autophagic flux (71). cAMP also modulates lysosomal pH and cathepsin D levels in human fibroblasts with the PS1-fAD mutation and in cultured cortical astrocytes by activating PKA (72–74). Increased cAMP and PKA activation recruits the H^+^/K^+^-ATPase/ZnT3 complex to lysosomes, which functions as a proton pump and modulates lysosomal pH and autophagy flux (75). PKA also modulates lysosomal-triggered calcium release (76).

We show here that monocytes from hyperammonemic rats show reduced staining with LysoTracker and cathepsin L levels and increased levels of LAMP2, indicating altered lysosomal and autophagy function. These effects are reversed by treatment with anti-TNFα or with an inhibitor of PKA and are reproduced in monocytes from control rats by addition of recombinant TNFα or of forskolin. This supports that the increase in cAMP and PKA activity induces alterations in the autophagy process and lysosomal function in monocytes from hyperammonemic rats, which would mediate the increase in EV release.

Several studies show that lysosomal and autophagy dysfunction enhances EV release. In astrocytes overexpressing α-synuclein, reduced LysoTracker staining and cathepsin L levels indicate lysosomal impairment, which leads to decreased degradation of multivesicular bodies (MVBs) and increased EV secretion (38). Monocytes from hyperammonemic rats exhibit similar lysosomal impairments, suggesting a comparable mechanism. Other studies also support that lysosomal dysfunction enhances EV release (39, 40). Gleason et al. (40) reported that lysosomal dysfunction enhances exocytosis, and hence, in lysosomal disorders, exosomal secretion may play a role in disease pathogenesis. Fussi et al. (39) show that α-synuclein increases LAMP2 and conclude that impaired formation of autophagosomes enhances the release of EV. Impairment of autophagosome–lysosome fusion has also been shown to increase the release of EV (77, 78).

The mechanism by which increased cAMP-PKA activity in hyperammonemia impairs autophagy and lysosomal function, enhancing EV release, would involve LC3. LC3 is a central protein in autophagy and autophagosome biogenesis and also mediates a pathway that promotes EV secretion, known as LC3-dependent extracellular vesicle loading and secretion (79–84). LC3 regulates MVB acidification by disrupting the V1V0-ATPase, increasing MVB pH, which is associated with enhanced EV release; similar effects have been shown with endolysosomal pH elevation using chloroquine or NH_4_Cl (84). LC3 phosphorylation by PKA reduces its recruitment to autophagosomes and autophagy induction, thus modulating EV secretion (85, 86).

We show that hyperammonemia reduces LC3 content in monocytes, and this is associated with autophagy-lysosomal dysfunction, increased lysosomal pH, and increased EV release. Reducing cAMP levels with anti-TNFα or PKA activity with an inhibitor prevents all these effects. Moreover, increasing cAMP in monocytes from control rats with recombinant TNFα or forskolin also reduces LC3 content and induces autophagy-lysosomal dysfunction, increased lysosomal pH, and increased EV release.

## 5 Conclusions

In summary, we show that in hyperammonemic rats, monocytes but not CD4^+^ lymphocytes release pathological EV. Hyperammonemia also increases the amount of EV released by monocytes and the content of TNFR1 and TNFα in the pathological EV, which induce glial activation, activation of the TNFα-TNFR1-S1PR2-IL-1β-IL-1 receptor-CCL2-BDNF-TrkB pathway, and alterations in the membrane expression of NMDA or AMPA receptors in hippocampal slices from control rats. These changes are responsible for the induction of cognitive impairment by EV from hyperammonemic rats (26, 36).

Both the increase of the amount of EV and the changes in the cargo of the EV with increased TNFR1 and TNFα in monocytes from hyperammonemic rats are a consequence of increased TNFα levels, which increase cAMP levels and PKA activity and reduce LC3 content (**Figure 12**). This leads to autophagy–lysosome dysfunction, with altered content of cathepsin L and LAMP2 and pH that leads to the increase in the release of EV and in their TNFR1 and TNFα content. All these changes are reversed by blocking TNFα with anti-TNFα or inhibiting PKA with an inhibitor. These data unveil the cell type that produces the pathological EV in hyperammonemia and the underlying mechanisms. As peripheral EV play a key role in triggering cognitive and motor impairment in hyperammonemia and minimal hepatic encephalopathy (24–27, 36), the data reported provide the bases for new treatments to improve cognitive and motor function in hyperammonemia and hepatic encephalopathy.

## Data Availability

The raw data supporting the conclusions of this article will be made available by the authors, without undue reservation.
